# Development of a tertiary lymphoid structure-based prognostic model for breast cancer: integrating single-cell sequencing and machine learning to enhance patient outcomes

**DOI:** 10.3389/fimmu.2025.1534928

**Published:** 2025-02-26

**Authors:** Xiaonan Zhang, Li Li, Xiaoyu Shi, Yunxia Zhao, Zhaogen Cai, Ni Ni, Di Yang, Zixin Meng, Xu Gao, Li Huang, Tao Wang

**Affiliations:** ^1^ Department of Pathophysiology, Bengbu Medical University, Bengbu, Anhui, China; ^2^ Department of Pathology, Bengbu Medical University, Bengbu, Anhui, China; ^3^ School of Clinical Medicine, Bengbu Medical University, Bengbu, Anhui, China; ^4^ School of Health Administration, Bengbu Medical University, Bengbu, Anhui, China; ^5^ Research Laboratory Center, Guizhou Provincial People’s Hospital, Guiyang, Guizhou, China

**Keywords:** breast cancer, tertiary lymphoid structures, machine learning algorithms, prognostic prediction models, immune microenvironment

## Abstract

**Background:**

Breast cancer, a highly prevalent global cancer, poses significant challenges, especially in advanced stages. Prognostic models are crucial to enhance patient outcomes. Tertiary lymphoid structures (TLS) within the tumor microenvironment have been associated with better prognostic outcomes.

**Methods:**

We analyzed data from 13 independent breast cancer cohorts, totaling over 9,551 patients. Using single-cell RNA sequencing and machine learning algorithms, we identified critical TLS-associated genes and developed a TLS-based predictive model. This model stratified patients into high and low-risk groups. Genomic alterations, immune infiltration, and cellular interactions within the tumor microenvironment were assessed.

**Results:**

The TLS-based model demonstrated superior accuracy compared to traditional models, predicting overall survival. High TLS patients had higher tumor mutation burden and more chromosomal alterations, correlating with poorer prognosis. High-risk patients exhibited a significant depletion of CD4^+^ T cells, CD8^+^ T cells, and B cells, as evidenced by single-cell and bulk transcriptomic analyses. In contrast, immune checkpoint inhibitors demonstrated greater efficacy in low-risk patients, whereas chemotherapy proved more effective for high-risk individuals.

**Conclusions:**

The TLS-based prognostic model is a robust tool for predicting breast cancer outcomes, highlighting the tumor microenvironment’s role in cancer progression. It enhances our understanding of breast cancer biology and supports personalized therapeutic strategies.

## Introduction

Breast cancer (BC) is a leading global cancer (1). Despite improvements in early diagnosis and treatment, managing advanced BC remains challenging (2). Current treatment options have achieved limited success, particularly in advanced stages (3). Thus, effective and accurate prognostic models are urgently needed to improve prognosis and treatment strategies for BC patients.

The pathogenesis of BC is complex, involving not only cancer cells but also the surrounding stromal cells (4). Recent research has shifted focus from solely targeting cancer cells to also considering the tumor microenvironment (5, 6). One area of interest is the study of tertiary lymphoid structures (TLS), which are ectopic lymphoid tissues found in the stroma of BC tissues (7). TLS can stimulate and promote immune responses against tumors by breaking immune tolerance or neglect (8). Understanding the role of TLS in BC could lead to novel therapeutic approaches.

Recent studies have shown that the presence of TLS in various cancers, including BC, is associated with better prognostic outcomes (4). TLSs are known to enhance anti-tumor immune responses by facilitating the activation and proliferation of T and B lymphocytes within the tumor microenvironment (9). In BC, higher densities of TLS have been correlated with improved survival rates and a better response to therapies, including immunotherapy (10). This suggests that TLS can serve as a prognostic marker and a potential therapeutic target.

This study seeks to thoroughly explore the role of TLS in BC progression. Employing advanced machine learning techniques, we pinpointed four key genes linked to TLS in BC lesions, forming the foundation for a predictive model. The model effectively categorized BC patients into high- and low-risk groups, using TLS-based nomograms to estimate overall survival (OS) across various time points. Our findings highlight the exceptional performance of the TLS-based predictive model in evaluating prognosis, immune profiles, and responses to immune checkpoint inhibitors (ICIs) and chemotherapy. Furthermore, the model successfully pinpointed novel therapeutic targets and drugs for BC patients. These results emphasize the critical role of TLS within the tumor microenvironment and its potential to enhance BC management and treatment outcomes. By tailoring therapeutic strategies to individual immune landscapes, this model marks a notable progression in personalized medicine.

## Materials and methods

### Data collection

Data were gathered from 15 separate breast cancer cohorts obtained from The Cancer Genome Atlas (TCGA), the Gene Expression Omnibus (GEO), and MetaGxData. Only those samples with complete survival data were chosen for analysis. A total of 9,551 patients were analyzed, representing cohorts including TCGA-BRCA (n = 1,076), GSE202203 (n = 3,206), GSE96058 (n = 3,409), GSE20685 (n = 327), GSE58812 (n = 107), GSE21653 (n = 244), GSE7390 (n = 198), GSE11121 (n = 200), GSE86166 (n = 330), GSE48391 (n = 81), GSE20711 (n = 88), PNC (n = 87), and TRANSBIG (n = 198).

### Machine learning derived signature

A total of ten computational tools, including RSF, LASSO, GBM, Survival-SVM, SuperPC, Ridge Regression, plsRcox, CoxBoost, Stepwise Cox, and Elastic Net (Enet), were employed in this study. Specifically, we have detailed the ten machine learning algorithms used to develop the TLS-based predictive model, including:

Random Survival Forest (RSF): A robust ensemble method that improves predictive performance by aggregating multiple decision trees trained on survival data. RSF is particularly effective in handling high-dimensional data and identifying complex interactions.

Least Absolute Shrinkage and Selection Operator (LASSO): A regression method that applies L1 regularization to select the most relevant features by shrinking the coefficients of less important variables to zero, thereby reducing overfitting.

Gradient Boosting Machine (GBM): An iterative learning technique that builds predictive models sequentially by minimizing residual errors, making it highly effective for structured data and survival analysis.

Survival Support Vector Machine (Survival-SVM): A technique that finds an optimal hyperplane for classification while considering censored survival data, ensuring robust patient stratification.

Supervised Principal Component (SuperPC): A method that identifies significant principal components associated with survival outcomes, improving interpretability and feature dimensionality reduction.

Ridge Regression: An L2 regularization technique that minimizes the impact of multicollinearity among features while ensuring model stability.

Partial Least Squares Cox Regression (plsRcox): A statistical method that models survival data by reducing dimensionality while capturing latent structures within the dataset.

CoxBoost: A boosting algorithm tailored for Cox proportional hazards models, allowing efficient handling of high-dimensional datasets with minimal overfitting.

Stepwise Cox Regression: A systematic method for feature selection in Cox regression that iteratively adds or removes variables based on statistical significance.

Elastic Net (Enet): A hybrid approach combining LASSO and Ridge regression to achieve both variable selection and model stability, offering improved generalization.

Among these tools, RSF, LASSO, CoxBoost, and Stepwise Cox were chosen due to their effectiveness in dimensionality reduction and variable selection. These techniques were combined into 108 different configurations to create a predictive signature, with performance assessed across all cohorts, which included both TCGA training and validation datasets. The most dependable prognostic model was determined by evaluating the average concordance index (C-index).

To further refine our model and ensure it included only the most predictive genes, we employed exhaustive search. This method evaluated all possible combinations of the selected genes to identify the subset that provided the best model performance based on predefined criteria. This step reduced the number of genes, focusing on those with the highest prognostic value.

Finally, a risk score for each patient was calculated using the expression levels of selected genes weighted by regression coefficients. This signature was validated across multiple independent cohorts to predict BC outcomes reliably.

### Genomic alteration analysis

Genetic differences between AITS groups were examined by evaluating mutation levels and Copy Number Alterations (CNA) using TCGA-BRCA data, providing crucial insights into cancer progression, tumor behavior, and therapeutic targets. Tumor Mutation Burden (TMB) was calculated for high and low AITS BC patients based on raw mutation files. TMB, reflecting the total mutations within a tumor genome, is linked to immunotherapy response, as higher TMB levels can generate neoantigens that stimulate immune reactions.

The maftools package was employed to visualize the most frequently mutated genes (mutation rate > 5%), offering a comprehensive view of common genetic alterations. Patient-specific mutational signatures were further analyzed using the deconstructSigs package, which interprets DNA damage and repair processes in cancer cells, shedding light on mutagenesis mechanisms (11). Four dominant mutational signatures—SBS2, SBS13, SBS7B, and SBS7D—were identified within the TCGA-BRCA dataset, highlighting distinct patterns of genomic instability associated with breast cancer.

Five common regions of amplification and deletion, crucial for understanding BC’s genomic landscape, were identified. Amplifications and deletions activate oncogenes or lead to the loss of tumor suppressor genes. Focus was given to four key genes in chromosomal regions 8q24.21 and 12p13.1, known for harboring vital oncogenes and tumor suppressor genes contributing to BC pathogenesis.

### Single-cell data processing

To prepare the dataset for analysis using single-cell RNA sequencing (scRNA-seq), we employed Seurat (v4.0) to process data sourced from GSE161529 (12). This method allows for a detailed investigation of cellular variations within tumors, which is crucial for comprehending the complex biology of cancer. We excluded genes that exhibited no expression, concentrating instead on those with detectable expression levels. The ‘SCTransform’ function in Seurat normalized the expression matrix to account for technical biases. Data dimensionality reduction was achieved through Principal Component Analysis (PCA) and Uniform Manifold Approximation and Projection (UMAP). PCA effectively maintains the majority of variability while simplifying the dataset, whereas UMAP offers a two-dimensional representation that captures the local structure of the data.Cellular populations were discerned using Seurat’s “FindNeighbors” and “FindClusters” functionalities. These methods construct a shared nearest neighbor graph to identify clusters of comparable cells. To enhance the dataset’s accuracy, the DoubletFinder package was utilized to remove potential doublets—artificial multiplets that may arise during sequencing (13). Rigorous quality control protocols were implemented; cells with mitochondrial gene composition exceeding 15% or those with fewer than 500 expressed genes were excluded. High mitochondrial levels may imply cellular stress or death, while low gene counts could indicate subpar cell quality. Consequently, these measures led to the generation of a dataset comprising 37,265 cells for subsequent analysis.Cell types were classified through manual annotation based on the identification of known marker genes, facilitating accurate categorization of the diverse cell populations present in the breast cancer samples.

### Inference of regulons and their activity

Single-Cell Regulatory Network Inference (SCENIC) was utilized to construct gene regulatory networks (GRNs) using single-cell RNA sequencing data (14). This framework involves a three-phase process to deduce regulons and evaluate their activities: First, co-expression modules were established through the clustering of genes exhibiting similar expression profiles, thereby highlighting possible regulatory interactions between transcription factors (TFs) and their target genes. This initial phase establishes the foundation for characterizing gene regulatory connections. Next, the identification of direct target genes associated with each co-expression module was conducted by examining the enrichment of TF motifs in the promoters of the co-expressed genes. Only target genes that demonstrated a significant enrichment of motifs corresponding to the relevant TFs were chosen, thereby refining the co-expression modules into regulons, each comprised of a TF and its associated direct targets. Finally, the regulatory activity of each regulon was quantified by computing the Regulatory Activity Score (RAS) for individual cells. This score was derived from assessing the area under the recovery curve, which indicates the level of activity of the regulon across different cells (15). To address the challenges related to the scalability of conventional SCENIC methodologies in handling large datasets and their susceptibility to variations in sequencing depth, the data were organized into metacells—aggregates of similar cells—before conducting SCENIC analysis. This adjustment greatly improved scalability, resilience, and data integrity while minimizing computational demands, rendering SCENIC more feasible for the examination of extensive single-cell RNA-seq datasets.

### Regulon clustering

A comprehensive computational system was developed to delineate the regulatory interactions among transcription factors (TFs) and their corresponding target genes, particularly emphasizing the clustering of TFs. This procedure encompassed several essential stages.

#### Filtering interaction pairs

TF-target interaction pairs were filtered to include only those exceeding a predefined significance threshold (>1), ensuring that the analysis concentrated on the most impactful regulatory interactions and enhancing result reliability.

#### Identifying key regulatory TFs

Pivotal TFs were identified based on the extent of their regulatory influence over target genes. These TFs, acting as hub genes, were subjected to in-depth analysis to understand their central roles within the regulatory network.

#### Creating an undirected graph model

An undirected graph was constructed to illustrate the intricate network of TF-target interactions. To enhance the spatial arrangement of the graph, a force-directed algorithm was utilized, effectively visualizing the structure of the network and the dynamic relationships between TFs and targets.

#### Community detection using the Leiden algorithm

Communities within the network were identified through the application of the Leiden algorithm, revealing the modular structure of TFs according to their regulatory connections. Each TF was allocated to a distinct cluster, allowing for a detailed examination of the regulatory landscape. By synthesizing these methods, the procedure offered a thorough perspective on regulatory networks, revealing complex interactions between TFs and their targets while identifying essential regulatory clusters for further investigation.

### Cell-cell communication analysis

The R package CellChat was employed to investigate cell-cell communication through the use of UMI count matrices for each experimental group (16). For the analysis of ligand-receptor interactions, the CellChatDB. human database was used as a reference, with the default settings of the package being applied consistently. In order to assess interaction counts and their respective intensities, CellChat objects from various groups were combined using the mergeCellChat function. The differences in both the number and strength of interactions between cell types were illustrated using netVisual_diffInteraction, while alterations in signaling pathways were identified with the rankNet function. Furthermore, the expression patterns of signaling genes were visualized through netVisual_bubble and netVisual_aggregate. Additionally, the NicheNet package was utilized to investigate intercellular communication by analyzing ligand activity and the expression patterns of downstream targets (17). This approach provided an extensive perspective on the signaling mechanisms that govern interactions among cell types, using ligand-target relationship data to deduce communication pathways within the cellular microenvironment.

### Evaluation of the tumor microenvironment and immunotherapy response

To comprehensively evaluate immune cell infiltration, multiple algorithms were employed to analyze the abundance and composition of infiltrating immune cells in AITS-classified patients (18). These included MCPcounter, EPIC, xCell, CIBERSORT, quanTIseq, and TIMER, each offering distinct insights into the tumor microenvironment (TME).

The TIDE index was calculated to provide a detailed representation of the immune landscape within the TME, predicting patient responses to immune checkpoint inhibitors (ICIs) and shedding light on the prognostic relevance for BC patients (19).

Immune checkpoints were assessed as key indicators of the immune state, facilitating preliminary predictions of patient responsiveness to ICI therapies. These analyses offered critical insights into the tumor’s immune environment, aiding in the evaluation of immunotherapy efficacy.

This integrative approach to profiling the immune landscape within the TME is pivotal for advancing personalized medicine, enabling the development of tailored treatment strategies that align with the unique immune characteristics of each patient.

### Determination of therapeutic targets and drugs for high AITS patients

To determine potential therapeutic targets and medications for patients with high AITS, compounds that were duplicates were excluded from the Drug Repurposing Hub, leading to a total of 6,125 unique compounds. Therapeutic targets relevant to breast cancer outcomes were determined using Spearman correlation analysis, which focused on the association between AITS and gene expression levels. Genes exhibiting a correlation coefficient greater than 0.15 along with a P-value of less than 0.05 were included, while those with a correlation coefficient lower than -0.30 and a P-value of less than 0.05 were associated with unfavorable prognosis. The relevance of these genes was further evaluated by investigating the correlation between CERES scores from the Cancer Cell Line Encyclopedia (CCLE) and risk scores specific to breast cells, thereby identifying genes crucial for the survival of cancer cells as potential therapeutic targets (20).

To enhance predictions regarding drug responsiveness, information from the Cancer Therapeutics Response Portal (CTRP) and the PRISM project was utilized. These platforms offer comprehensive drug screening and molecular data across a range of cancer cell lines. A differential expression analysis was performed between bulk samples and cell line data, and the pRRophetic package was utilized to apply a ridge regression model for predicting drug response. This model, which was developed using expression data and drug response information from solid Cancer Cell Lines (CCLs), demonstrated exceptional predictive accuracy, validated through a 10-fold cross-validation process (21).

Furthermore, to pinpoint promising therapeutic agents for breast cancer, an analysis using the Connectivity Map (CMap) was conducted. This process involved comparing gene expression profiles across various risk subgroups and submitting the top 300 genes (150 that were up-regulated and 150 that were down-regulated) to the CMap database. A negative CMap score suggested an increased therapeutic potential against breast cancer, indicating an inverse correlation between the CMap score and the efficacy of a compound.

### Patient stratification

In our study, patient stratification was performed based on the expression levels of key genes identified by our TLS-based prognostic model. First, RNA was isolated from BC specimens by employing TRIzol reagent (Invitrogen). The synthesis of complementary DNA (cDNA) was carried out using GoScript reverse transcriptase, followed by qRT-PCR using Master Mix (Promega) according to the manufacturer’s instructions. Data acquisition was performed with the CFX96 Touch Real-Time PCR Detection System (BioRad). Quantification of gene expression was executed through the 2^-ΔΔCq^ method, utilizing GAPDH for normalization. A patient-specific risk score was then computed based on the expression levels of the selected TLS-related genes, weighted by their regression coefficients derived from the machine learning model (AITS). This categorization allowed for the identification of patients exhibiting diverse risk profiles, thereby aiding in the formulation of customized therapeutic strategies.

### Immunohistochemistry experiment

Tissue specimens were obtained from 30 patients with breast cancer who were undergoing surgical procedures at the Guizhou Provincial People’s Hospital. Following established protocols, these specimens underwent Hematoxylin and Eosin (H&E) staining, with diagnoses independently verified by two pathologists. In the analysis of immunohistochemistry (IHC), the procedures for samples embedded in paraffin were adhered to as described in earlier studies (22, 23). To evaluate protein expression levels, standardized protocols and scoring systems were utilized. The IHC outcomes were assessed independently by two pathologists to ensure alignment with methodologies from previous studies (23).

## Results

### Integrative construction of an artificial intelligence signature

**Figure 1:**
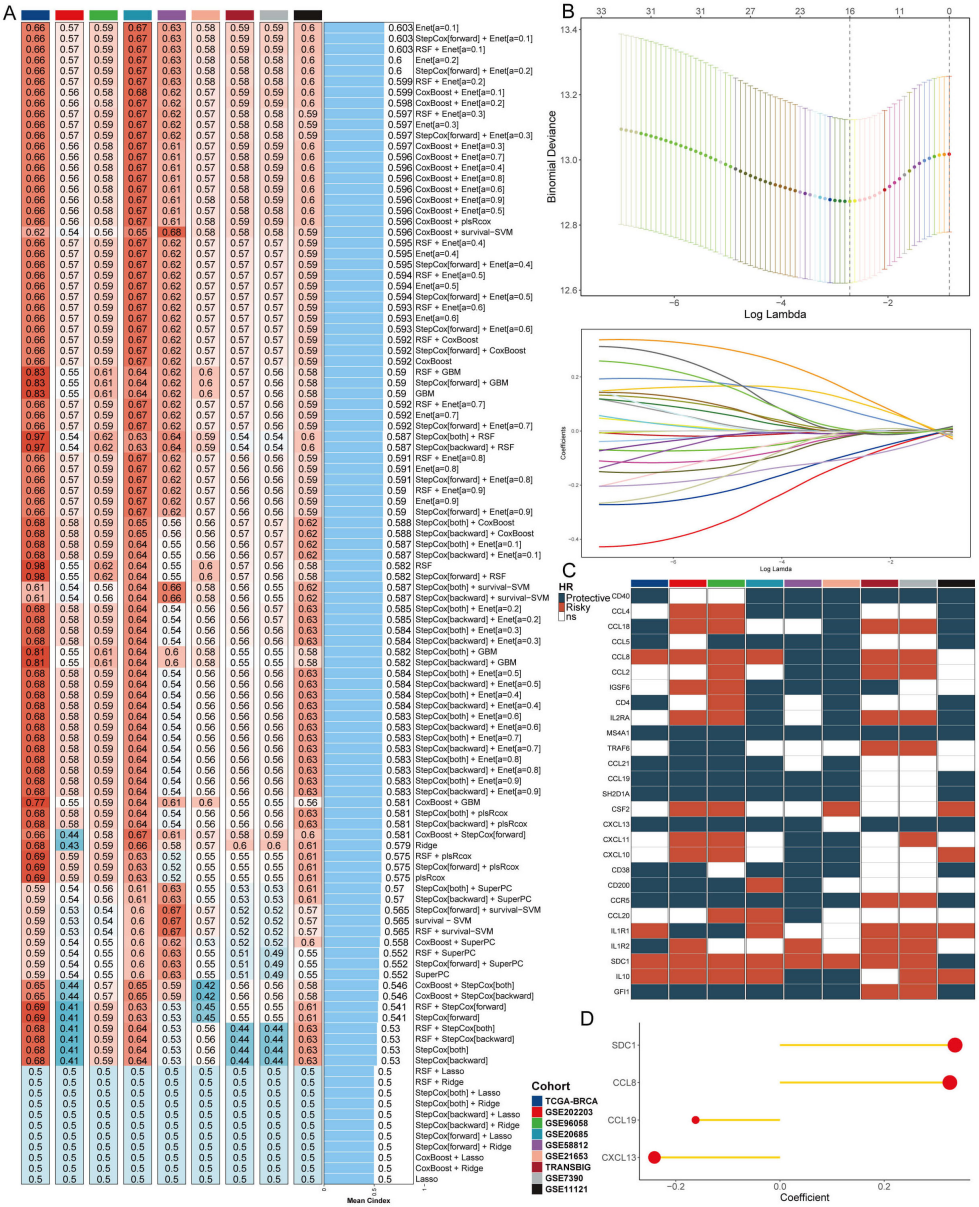
Development and validation of the artificial intelligence-assisted TLS signature. **(A)** Average C-index for each machine learning algorithm combination in the TCGA-BRCA training cohort and 8 validation cohorts. **(B)** Identification of 27 TLS genes contributing significantly to the model using Enet regression with 10-fold cross-validation. **(C)** Prognostic significance of the 27 TLS genes evaluated across multiple datasets using univariate Cox regression. **(D)** Final selection of 4 TLS genes based on an exhaustive search, with patient risk scores calculated according to the expression levels of these genes and their regression coefficients.

To comprehensively investigate the clinical relevance of TLS in BC, we developed an artificial intelligence-assisted TLS signature (AITS) by utilizing 10 machine learning algorithms across 108 combinations. In the TCGA-BRCA training cohort, along with 8 validation cohorts, we calculated the average C-index for each algorithm combination to determine their predictive performance (**Figure 1A**). Among these, the Enet algorithm (α = 0.1) demonstrated the highest average C-index, establishing it as the optimal model (**Figure 1A**).

The Enet algorithm combines the properties of both Lasso and Ridge regressions to improve model accuracy and interpretability. Using Enet regression with 10-fold cross-validation, we identified 27 TLS genes that significantly contributed to the model (**Figure 1B**). These genes were further evaluated for their prognostic significance across multiple datasets using univariate Cox regression (**Figure 1C**). Univariate Cox regression helps in understanding the relationship between each gene’s expression level and patient survival, thus highlighting the most significant predictors.

An exhaustive search was then conducted to identify the most predictive subset of these genes. Exhaustive search involves evaluating all possible combinations of features to find the subset that offers the best predictive performance, ultimately selecting 4 TLS genes. Each patient’s risk score was subsequently calculated based on the expression levels of these 4 genes, weighted by their regression coefficients (**Figure 1D**). This approach ensures that the most relevant and impactful genes are included in the final model, enhancing its predictive power and clinical utility.

### Independent prognostic value of AITS

Patients were categorized into high-risk and low-risk groups by utilizing the survminer package, which facilitated the identification of optimal cutoff values. The implementation of the Kaplan-Meier survival analysis revealed a strikingly higher mortality rate among individuals classified in the high-risk group within the training cohort. Moreover, these trends were consistent in the validation cohorts, as illustrated in **Supplementary Figure S1A**. The performance of the AITS model in the training cohort (TCGA-BRCA) was notably robust, exhibiting time-dependent area under the curves (AUCs) of 0.659, 0.726, and 0.668 at the 1, 3, and 5-year marks, respectively. This strong performance was further corroborated by analogous outcomes in the validation cohorts, as depicted in **Supplementary Figure S1B**.

Univariate and multivariate Cox regression analyses were conducted on variables including age, menopause status, TNM stage, pathological stage, ER, PR, HER2 expression, and the AITS model to determine if the prognostic significance of AITS was independent of clinical traits and molecular features. The AITS model remained statistically significant for overall survival (OS) after adjusting for these variables, suggesting it as an independent risk factor in BC (**Supplementary Figure S2A**).

A nomogram that includes AITS, age, and pathological stage was created to forecast the survival probabilities for BC patients at one, three, and five years (**Supplementary Figure S2B**). The accuracy of the model was validated by the calibration curve, which indicated a strong alignment with actual survival rates (**Supplementary Figure S2C**). There were no significant differences (p > 0.05) between the values predicted by the AITS chart and the observed outcomes, reinforcing its predictive strength (**Supplementary Figure S2D**). The AITS chart demonstrated superior predictive performance compared to the extreme curves (Treat All and Treat None) (**Supplementary Figure S2E**). In contrast to other clinical pathological factors, the AITS model showed a greater reflection of prognostic correlation in BC (**Supplementary Figure S2F**).

### Comparative performance analysis of AITS and published gene signatures

Recent advancements in high-throughput sequencing and computational biology have led to the development of numerous predictive gene expression signatures through a variety of machine learning methodologies. In order to evaluate AITS performance against other signatures, we examined 100 published signatures generated using different algorithms.

**Figure 2:**
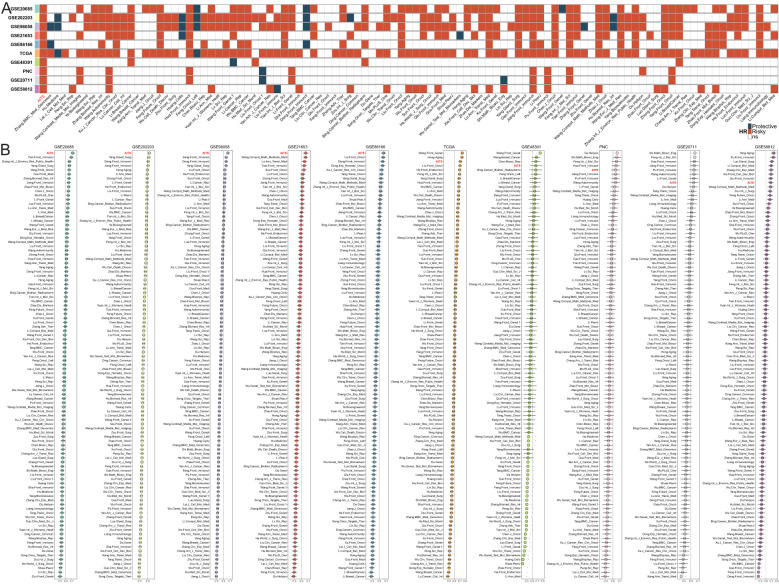
Comparison of AITS with 100 published signatures. **(A)** Univariate Cox regression analysis showing that the AITS model maintains complete significance across all datasets. **(B)** C-indices of all cohorts for each signature.

Univariate Cox regression analysis was used for each signature. The AITS model uniquely maintained complete significance across all datasets, highlighting its stability in predicting BC recurrence risk (**Figure 2A**). C-indices were calculated for each signature across all cohorts. Our findings revealed that the AITS model consistently achieved the highest predictive power in several cohorts, including GSE20685, GSE202203, GSE96058, GSE21653, and GSE86166, and also ranked first in the TCGA, GSE48391, PNC, GSE20711, and GSE58812 cohorts (**Figure 2B**). This highlights the superior predictive performance of the AITS model compared to nearly all other models in each cohort.

### Genome alterations and landscape of AITS

**Figure 3:**
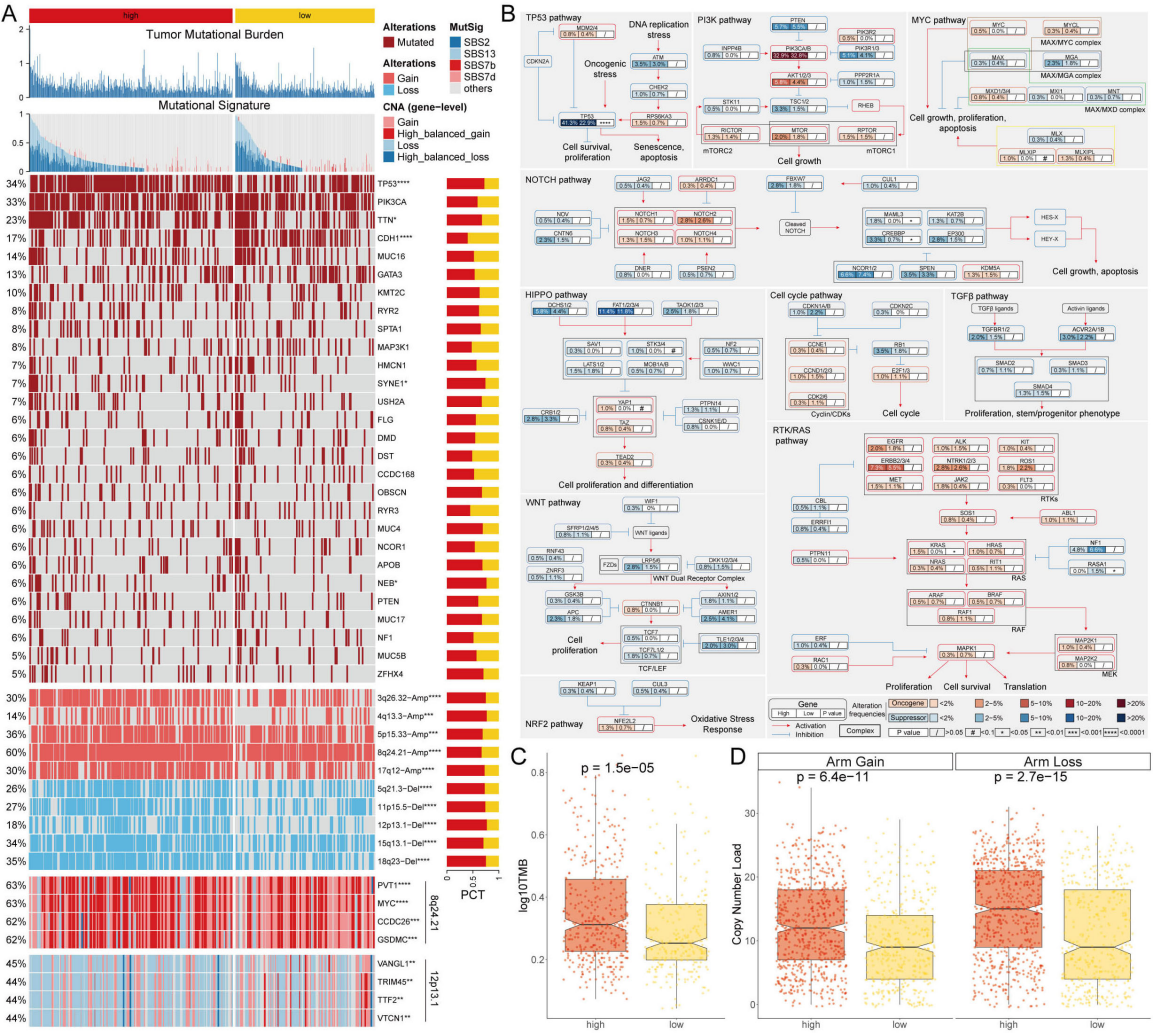
Genome alteration landscape of AITS. **(A)** Multi-omics analysis showing TMB, mutational signatures, gene mutations, and copy number variations. **(B)** Analysis of 10 oncogenic signaling pathways highlighting differential mutation frequencies between high and low AITS groups. **(C)** TMB analysis indicating significantly higher TMB in the high AITS group. **(D)** CNA landscape showing significant amplifications and deletions in high AITS group compared to low AITS group.

Genomic alterations were identified through multi-omics analysis, including assessments of tumor mutation burden (TMB), mutational signatures, gene mutations, and copy number variations (**Figure 3A**). An examination of ten oncogenic signaling pathways within the TCGA dataset demonstrated that tumor suppressor genes such as TP53, CREBBP, and RASA1 exhibited elevated mutation frequencies in the high AITS category. In contrast, mutations in CDH1, TTN, and KRAS were more prevalent in the low AITS category (**Figures 3A, B**). Additionally, the tumor mutation burden (TMB) was significantly greater in the high TLS category (**Figure 3C**).

Further analysis of the copy number alteration (CNA) landscape between the high and low AITS groups showed significantly more amplifications and deletions in the high AITS group. Key amplification regions included 3q26.32, 4q13.3, 5p15.33, 8q24.21, and 17q12, while significant deletions were noted at 5q21.3, 11p15.5, 12p13.1, 15q13.1, and 18q23 (**Figures 3A, D**). At the gene level, oncogenes such as PVT1, MYC, CCDC26, and GSDMC were notably amplified within 8q24.21, whereas VANGL, TRIM45, TTF2, and VTCN1 were significantly deleted within 12p13.1 (**Figure 3A**).

### Single-cell analysis of biological mechanisms underlying AITS

**Figure 4:**
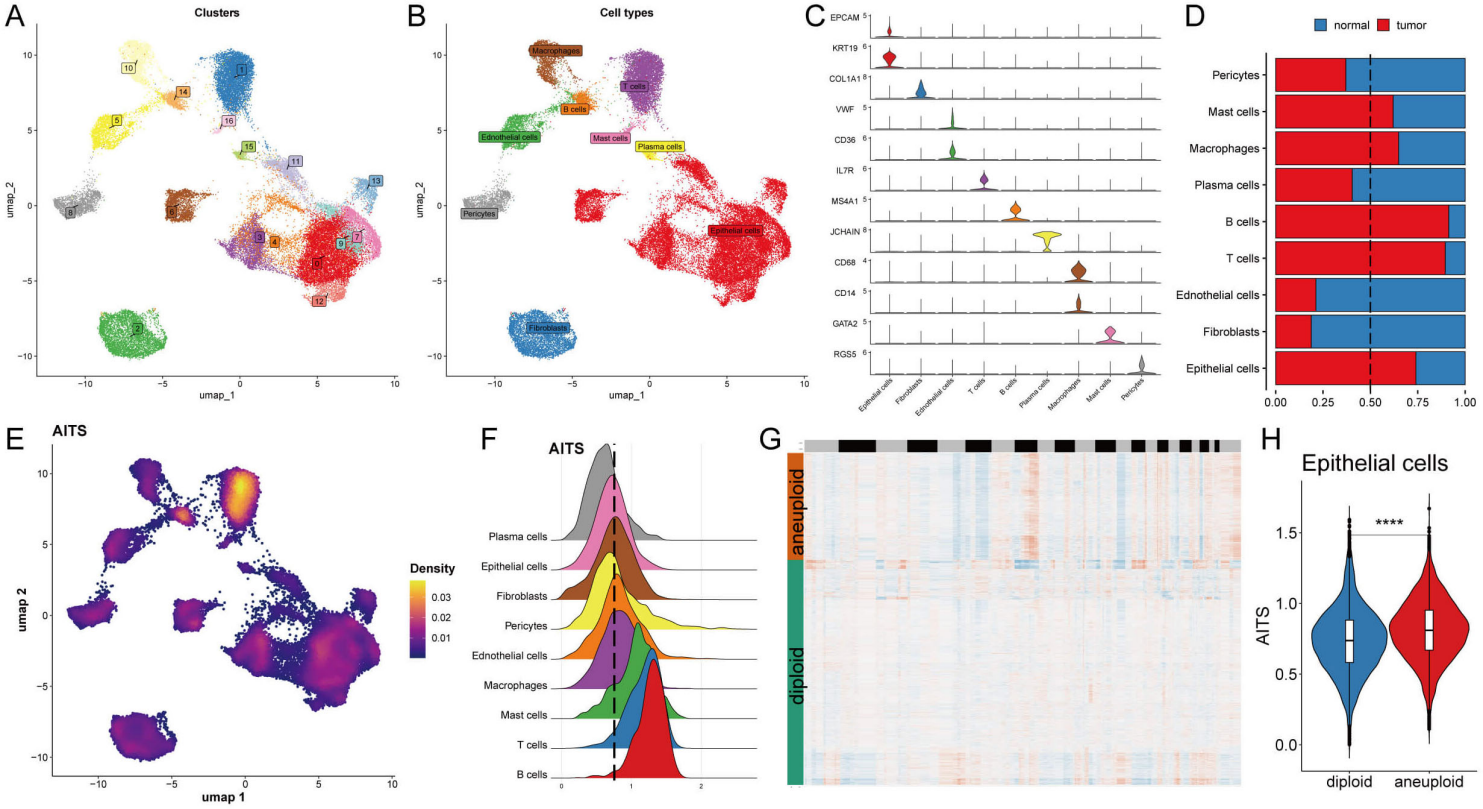
Potential biological mechanisms of AITS at the single-cell level. **(A)** Identification of seventeen clusters in single-cell transcriptome analysis. **(B)** Classification of nine cell types. **(C)** Representative markers for each cell type. **(D)** Distribution of cell types between tumor and normal tissues. **(E)** AITS scores across cells showing significant differences in distribution. **(F)** Grouping of cells based on epithelial cell peaks. **(G)** CopyKat algorithm analyzed the distribution of diploid and aneuploid cells. **(H)** Comparison of AITS scores between aneuploid and diploid epithelial cells. ^****^P<0.0001.

An analysis of the single-cell transcriptome was conducted to evaluate the AITS in eight patients with breast cancer, encompassing both tumor and adjacent normal tissues (**Supplementary Figures S3A, B**). A total of seventeen unique clusters and nine distinct cell types were recognized (**Figures 4A, B**). The quantities and proportions of each cell type were compiled across the patient cohort (**Supplementary Figures S3C, D**), along with representative markers illustrated for each cell type (**Figure 4C**, **Supplementary Figure S3E**).

A comparative analysis of cell type distribution between tumor and normal tissues revealed significant differences in the abundance of certain immune and epithelial cell types. Specifically, mast cells, macrophages, B cells, T cells, and epithelial cells were observed to be more prevalent in tumor tissues. Conversely, other cell types were found to be predominantly located in normal tissues, highlighting a notable variation in cellular composition between the two environments. This distinction emphasizes the potential role of specific immune cells in tumor progression and the unique microenvironment of tumors, as illustrated in **Figure 4D**. Each cell was then assigned an AITS score, revealing notable differences in cell distribution (**Figure 4E**). Cells were grouped according to their epithelial cell peaks (**Figure 4F**), and differential expression analysis combined with GSEA identified potential functional pathways linked to AITS (**Supplementary Figure S3F, G**). For epithelial cells from tumor samples, the high AITS group was associated with pathways involving ribosome and protein export, whereas the low AITS group was linked to spliceosome, protein processing in the endoplasmic reticulum, and proteasome pathways (**Supplementary Figure S3G**). Using the copyKat algorithm to further analyze tumor cells, it was shown that aneuploid epithelial cells had higher AITS scores compared to diploid tumor cells (**Figures 4G, H**).

### Transcriptional regulation and cell type-specific networks in AITS

**Figure 5:**
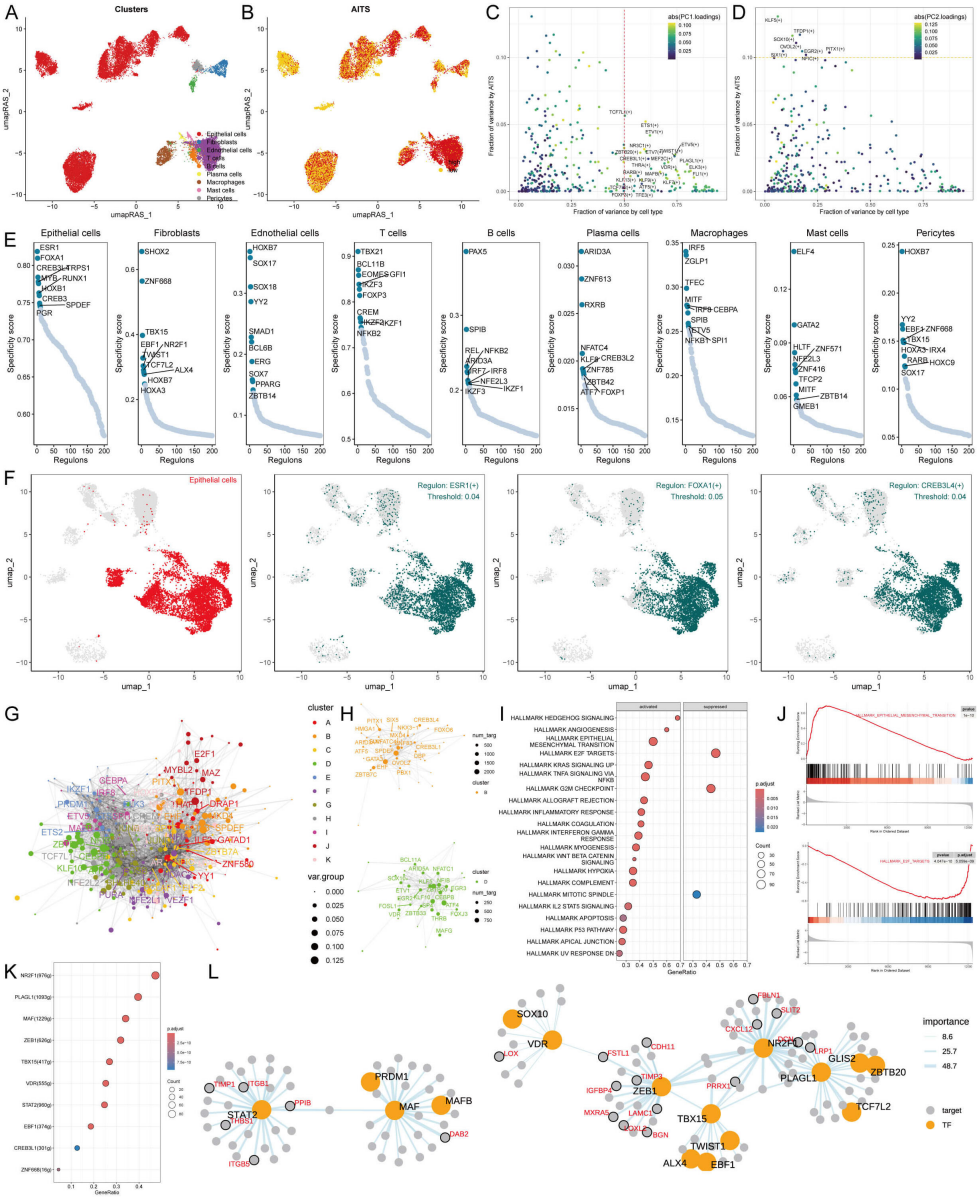
Transcriptional regulation of AITS and different cell types. **(A)** Clustering of cell types using UMAP. **(B)** SCENIC pipeline analysis translating gene expression data into RAS for transcription factors. **(C)** Variance decomposition using PCA to identify PC1 representing cell type-specific TFs. **(D)** PC2 representing AITS-specific TFs. **(E)** Regulon specificity scores (RSS) highlighting key regulators for different cell types. **(F)** UMAP plots showing specific regulators for epithelial cells. **(G)** Transcription factor interaction networks organized by RAS similarity using the Leiden algorithm. **(H)** Important transcription factor components in AITS. **(I)** GSEA results showing signaling pathway changes in high AITS epithelial cells. **(J)** Specific pathways like EMT activation and E2F target inhibition. **(K)** Identification of transcription factors contributing to EMT. **(L)** Network diagrams illustrating regulatory relationships among transcription factors.

To develop comprehensive gene regulatory networks for significant cell types, we utilized the SCENIC pipeline, which examines single-cell RNA sequencing data alongside cis-regulatory sequence information. This framework converts gene expression information into regulator activity scores (RAS) for transcription factors (TFs) (**Figures 5A, B**). Moreover, we conducted principal component analysis (PCA) for variance decomposition to distinguish unique regulons linked to AITS and cellular structures. The first principal component (PC1) predominantly highlighted TFs particular to distinct cell types, while the second principal component (PC2) was related to TFs unique to AITS (**Figures 5C, D**).

We identified crucial regulators that are vital for cell identity. We assessed the activity of each regulon associated with various cell types, deriving a regulon specificity score (RSS) through Jensen-Shannon divergence (**Figure 5E**). Regulators exhibiting the highest RSS scores were chosen for a deeper investigation into their functional characteristics. For epithelial cells, the most specific regulators identified were CREB3L4, SPDEF, and GATA3 (**Figure 5E**). This finding was also presented through UMAP plots (**Figure 5F**). Additionally, correlations between other cell types and their respective specific regulators were demonstrated (**Supplementary Figure S4A**).

Gene expression coordination often requires interactions among transcription factors. To systematically analyze the combination pattern of AITS, we compared RAS scores of each transcription factor using the Leiden algorithm. The results indicated that these transcription factors were organized into 11 components based on RAS similarity, with components B and D playing significant roles in AITS (**Figures 5G, H**, **Supplementary Figure S4B**).

Further exploration into the transcription factors driving AITS-related transcriptional changes in epithelial cells showed multiple signaling pathway alterations through GSEA (**Figure 5I**). For example, epithelial-mesenchymal transition (EMT) was activated in high AITS epithelial cells, while E2F targets were inhibited (**Figures 5I, J**). The transcription factors contributing to EMT were identified, and the regulatory relationships among these factors were visualized using network diagrams (**Figures 5K, L**).

### Intercellular communication patterns in AITS

**Figure 6:**
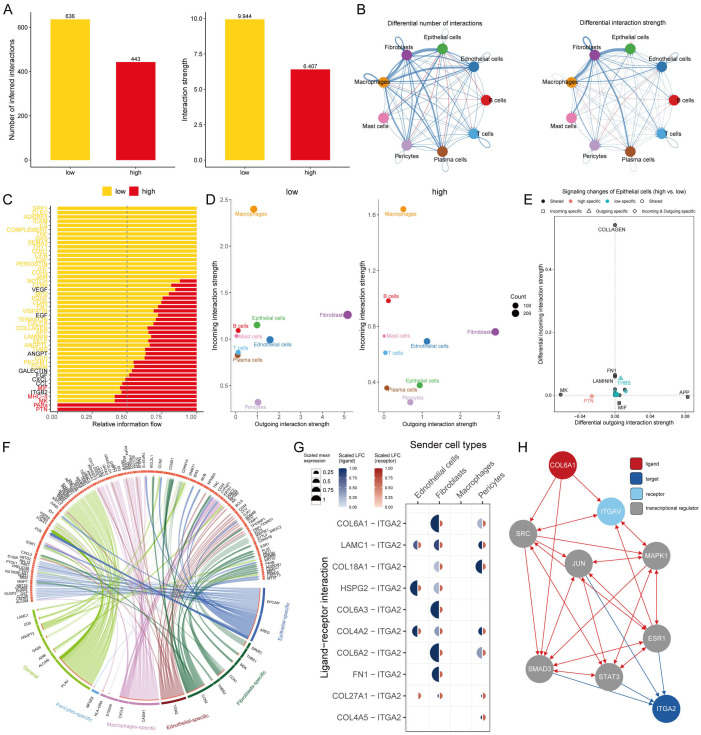
Intercellular communication in AITS. **(A)** Analysis of the quantity and strength of cell interactions showing reduced communication in the high AITS group. **(B)** Interaction network visualization of cell communication. **(C)** Comparison of 48 signaling pathways between the two groups. **(D)** Analysis of outgoing and incoming interaction intensity. **(E)** Specific pathways in epithelial cells related to AITS, such as PTN and THBS. **(F)** Circos diagram depicting significant ligand-receptor interactions. **(G)** Detailed interaction between ligand and receptor. **(H)** Ligand action network showing direct and indirect regulatory effects on target activity.

To understand the role of intercellular interactions in BC development, CellChat analysis was employed to evaluate interactions among AITS across nine different cell types. The analysis of cell interaction quantity and strength revealed reduced communication in the high AITS group (**Figure 6A**). A network visualization of these interactions indicated that in the high AITS group, epithelial cells exhibited enhanced interactions with various cell types such as T cells, B cells, mast cells, and plasma cells, whereas the interactions between T cells and B cells were notably weak (**Figure 6B**).

We compared 48 signaling pathways between the high and low AITS groups. Pathways such as PTN, PARs, MK, MHC-II, and MIF were predominantly active in high-AITS cells, whereas LAMININ, SPP1, CLEC, ADGRE5, and ICAM were more active in low-AITS cells (**Figure 6C**). The intensity of both outgoing and incoming interactions was also examined to observe cell dynamics. Epithelial cells in the high AITS group had weaker incoming interactions (**Figure 6D**). Several pathways in epithelial cells were specific to AITS, including PTN and THBS (**Figure 6E**).

Further analysis focused on the functions of various ligand-receptor pairs, presenting the key interactions in a circos diagram (**Figure 6F**). Notably, the COL6A1 ligand expressed on fibroblasts and pericytes bound to the ITGA2 receptor (**Figure 6G**). The ligand action network indicated that ligands could bind with other ligands to regulate downstream transcription factors, exerting both direct and indirect regulatory effects on targets (**Figure 6H**).

### Evaluating immunotherapy targets in the context of AITS

**Figure 7:**
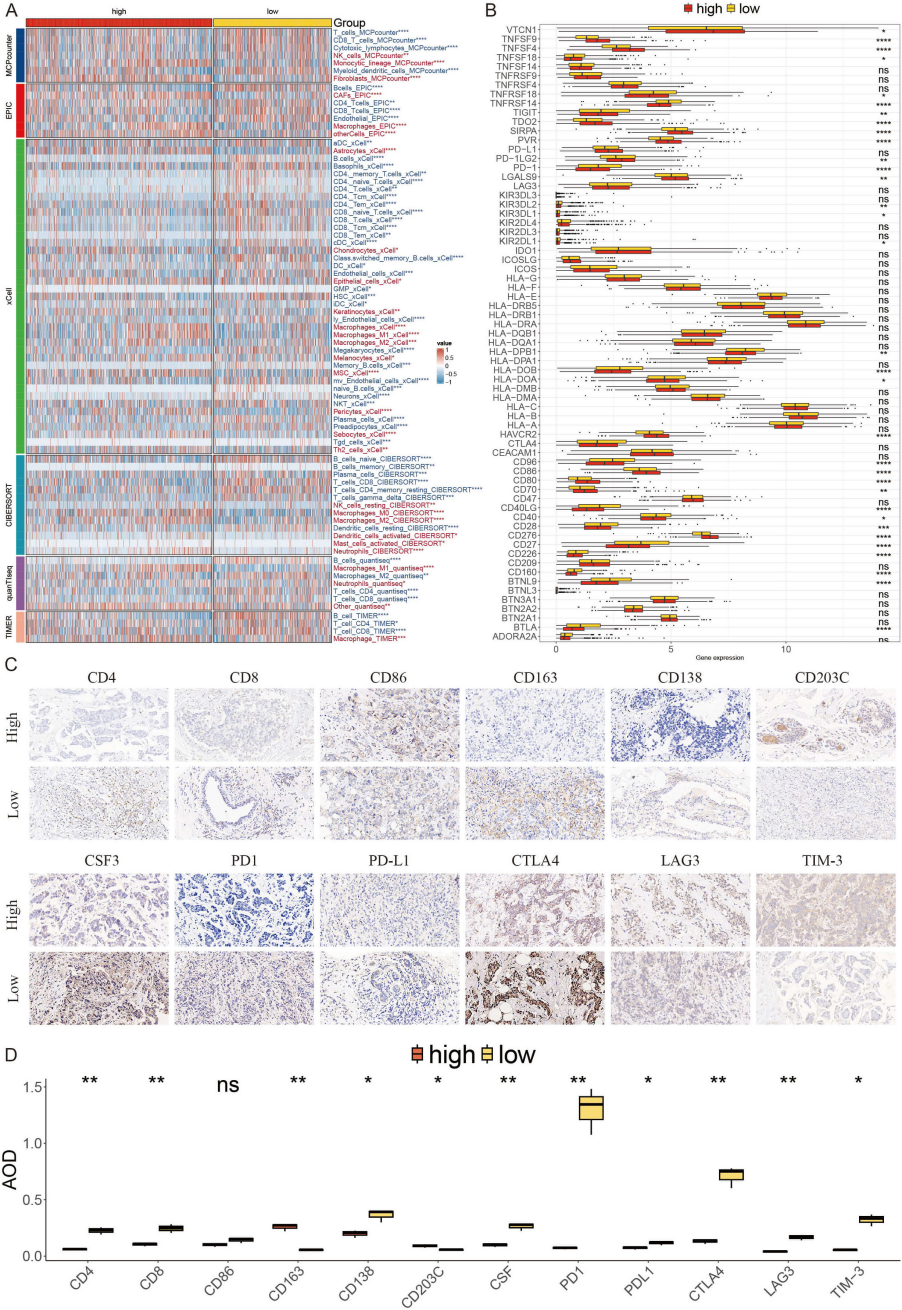
Differential expression and immunohistochemical analysis of immune markers in tumor microenvironments between AITS subgroups. **(A)** Heatmap providing a comparative view of immune cell infiltration in tumor samples with low and high AITS, utilizing various computational algorithms for quantification. Each row represents a different type of immune cell, with the color intensity reflecting the level of infiltration. Red text indicates increased infiltration in the high AITS group, while blue text indicates decreased infiltration. **(B)** Box plots illustrating the distribution of gene expression levels for ICIs across low versus high AITS conditions, with statistical significance denoted by ns for not significant; *P < 0.05; **P < 0.01; ***P < 0.001; ****P < 0.0001. **(C)** Representative immunohistochemistry images showcasing the staining intensity of various immune markers between high and low expression conditions, visually depicting the differential expression of these markers in correlation with AITS levels. **(D)** Box plots displaying the average optical density (AOD) of staining for immune markers, comparing high and low expression conditions, with statistical significance indicated by stars (* for p < 0.05, ** for p < 0.01, and ns for not significant).

Recognizing the crucial role of the immune microenvironment in tumor progression, we analyzed immune infiltration in BC patients using six different algorithms. The results showed reduced infiltration of CD4^+^ T cells, CD8^+^ T cells, and B cells in the high AITS group (**Figure 7A**). Additionally, there was increased expression of ICIs such as PD1, TIGIT, CTLA4, and members of the HLA family in the low AITS group (**Figure 7B**). These results were validated through immunohistochemistry (**Figures 7C, D**).

**Figure 8:**
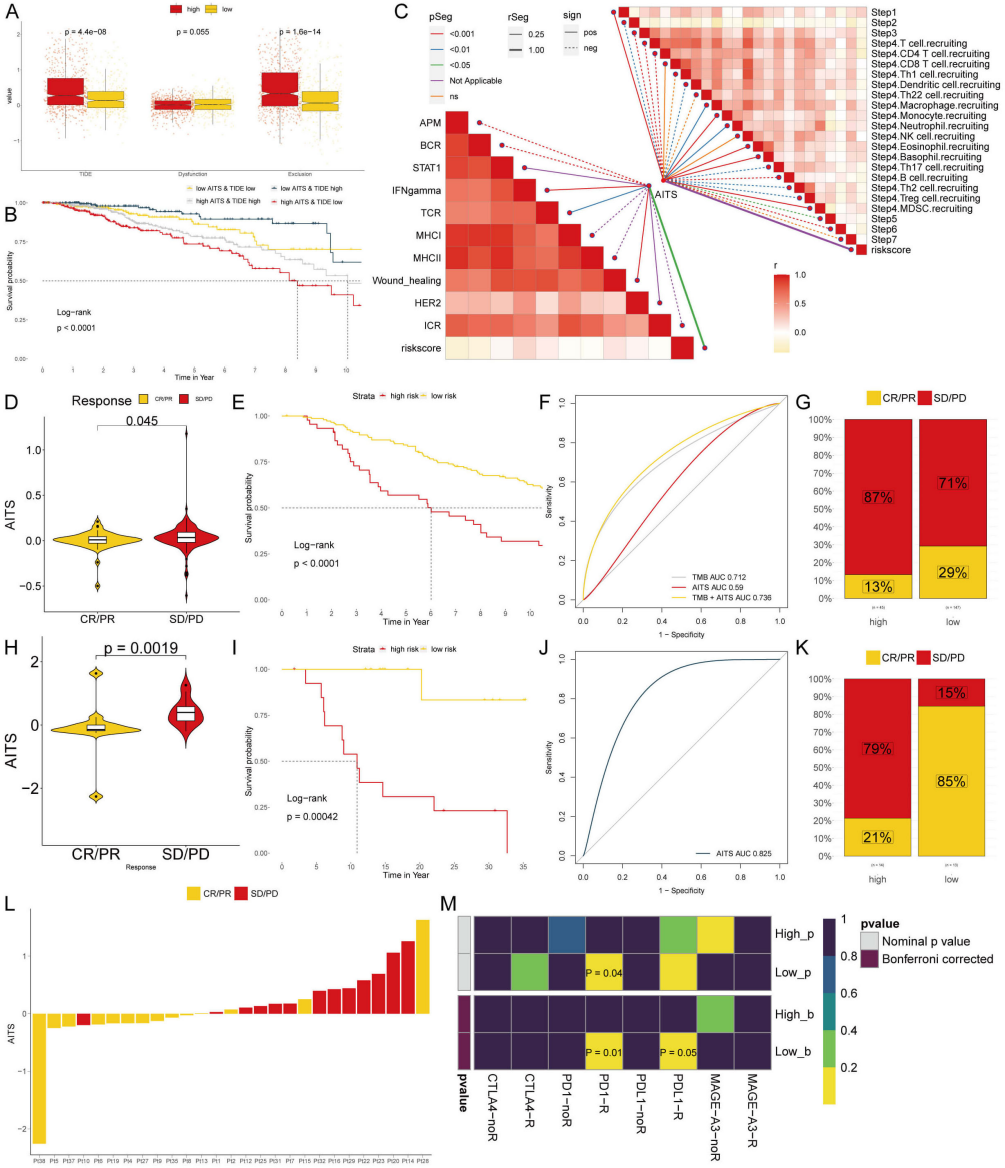
Analyzing potential immunotherapy targets for AITS. **(A)** Difference of TIDE, Dysfunction, Exclusion between the AITS groups. **(B)** The survival probability curves of four combinations of AITS and TIDE. **(C)** The correlation of AITS with 7 steps of tumor immune cycle and 10 signaling pathways related to tumor immunology. **(D, H)** Violin charts display the relationship between AITS levels and responses to anti-PDL1 **(D)** and anti-PD1 **(H)** therapies. **(E, I)** Survival probabilities of low and high AITS patients in anti-PDL1 **(E)** and anti-PD1 **(I)** cohorts, respectively, illustrating the impact of AITS on survival outcomes. **(F, J)** Analysis estimates the predictive ability of AITS via AUC values, considering TMB combinations, in anti-PDL1 **(F)** and anti-PD1 **(J)** cohorts, evaluating the efficacy of AITS as a biomarker. **(G, K)** The percentages of complete response/partial response (CR/PR) and stable disease/progressive disease (SD/PD) in anti-PDL1 **(G)** and anti-PD1 **(K)** cohorts are shown, based on AITS levels, to assess treatment effectiveness. **(L)** Distribution of AITS score of different patients after anti-PD1 treatment. **(M)** Heatmap demonstrating the predictive power of AITS for responsiveness to different ICIs treatment.

To examine differences in immunotherapy response between the groups, we evaluated patients using TIDE, dysfunction, and exclusion scores. Findings indicated that patients with higher AITS had elevated TIDE and exclusion scores, while dysfunction scores did not significantly differ within the TLS cohort (**Figure 8A**). Our analysis also revealed that patients with low AITS and high TIDE had more favorable outcomes compared to other groups (**Figure 8B**). Correlation analysis showed that anti-tumor immune activity was higher in low AITS patients compared to those with high AITS (**Figure 8C**).

Immune checkpoint inhibitors (ICIs) have revolutionized the field of cancer immunotherapy; however, their effectiveness in solid tumors such as breast cancer (BC) is still restricted. We evaluated the predictive significance of AITS levels in relation to immune checkpoint blockade therapies within the IMvigor210 (anti-PD-L1) and GSE78220 (anti-PD-1) groups. Patients with low AITS levels exhibited notable clinical advantages and improved survival rates when treated with anti-PD-L1 therapy (**Figures 8D–G**). Comparable advantages were noted for low AITS patients undergoing anti-PD-1 treatment (**Figures 8H–L**). By employing SubMap algorithms, we verified that patients with low AITS levels were considerably more inclined to experience benefits from both anti-PD-L1 and PD-1 therapies (**Figure 8M**). These results indicate that patients with low AITS levels may attain enhanced outcomes with ICI treatments.

### Identification of therapeutic agents for high AITS patients

**Figure 9:**
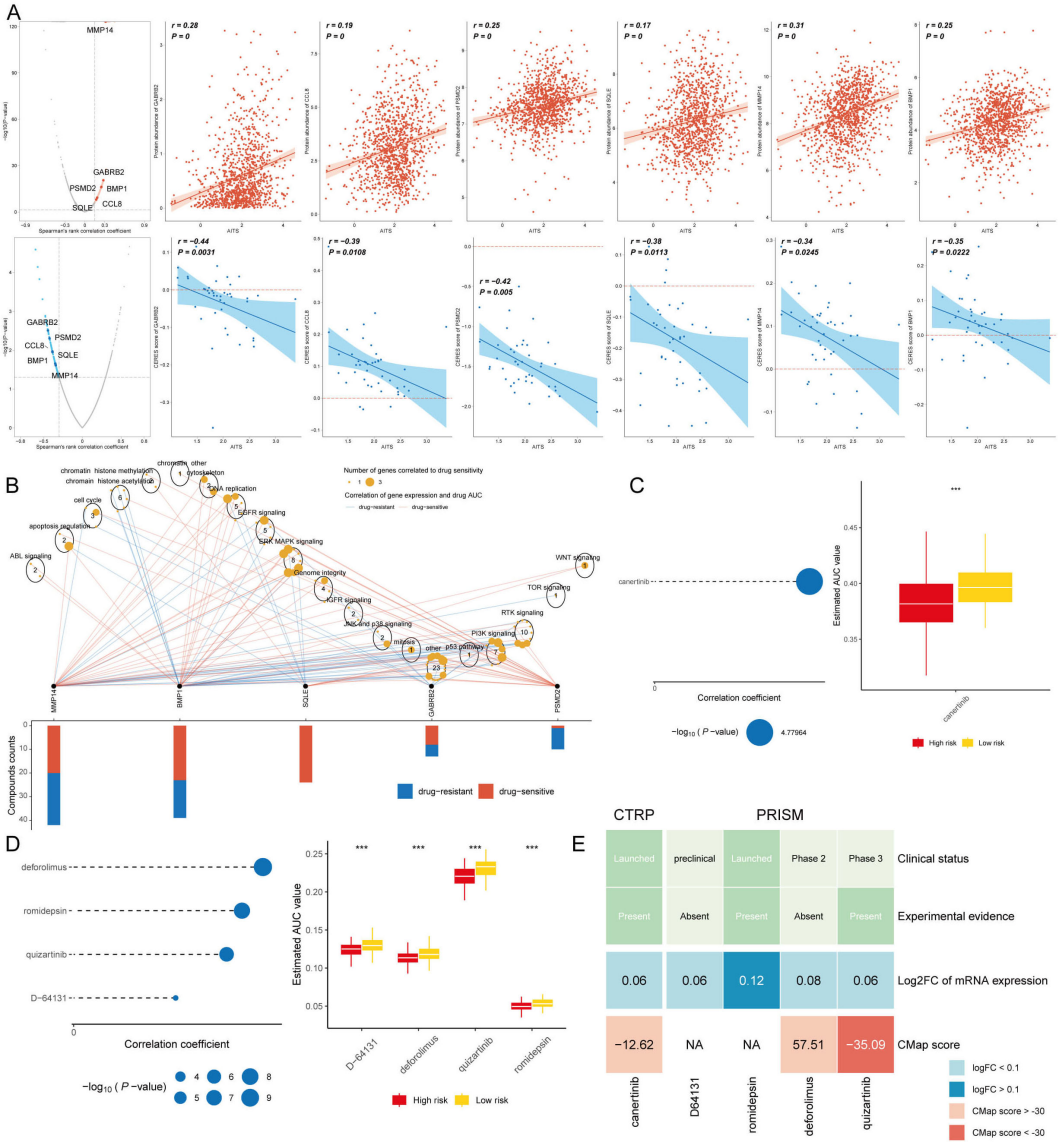
Identification of therapeutic agents for high AITS patients. **(A)** Spearman correlation analysis showing positive correlation of AITS with six targets (GABRB2, PSMD2, CCL8, BMP1, SQLE, MMP14) and significant negative correlation with CERES score. **(B)** Drug pathway analysis linking five targets (excluding GABRB2) to multiple drug pathways, highlighting their importance as therapeutic targets. **(C)** AUC values of identified compounds from CTRP database. **(D)** AUC values of identified compounds from PRISM database. **(E)** Analysis of clinical status, experimental evidence, mRNA expression levels, and CMap scores for selected compounds, with quizartinib identified as a potential therapeutic agent for high AITS patients.

Chemotherapy continues to be a key strategy for treating cancer, with information gathered from various datasets utilized to pinpoint possible medications for breast cancer patients exhibiting elevated AITS. Using Spearman correlation analysis, we found that AITS positively correlated with six targets (GABRB2, PSMD2, CCL8, BMP1, SQLE, MMP14) and had a significant negative correlation with CERES score, suggesting these targets as potential therapies for high AITS patients (**Figure 9A**). Five of these targets, excluding GABRB2, are linked to various drug pathways, making them key therapeutic targets for high AITS BC patients (**Figure 9B**).

From the CTPR and PRISM datasets, we identified five compounds: canertinib, deforolimus, romidepsin, quizartinib, and D-64131. A comparison of the AUC values of these compounds between patients with high and low AITS revealed that those with high AITS exhibited lower AUC values (**Figures 9C, D**). In our search for the most suitable therapeutic agent, we evaluated the clinical conditions, experimental data, mRNA expression levels, and CMap scores for each compound. According to the CMap score analysis, quizartinib emerged as a promising therapeutic candidate for patients with elevated AITS (**Figure 9E**).

## Discussion

In 2020, BC has become the most common cancer worldwide, particularly affecting women. It ranks first in cancer-related deaths, posing a serious threat to women’s health (24). Although significant progress has been made in diagnosis, surgery, and drug development, BC treatment still faces severe challenges due to inadequate treatment responses, recurrence, and metastasis (25, 26). Therefore, improving the therapeutic effectiveness of BC is crucial. Recent advancements in machine learning algorithms have enabled the construction of predictive models that enhance the accuracy of BC treatment selection.

TLS are ectopic lymphoid tissues found in non-lymphoid tissues. TLS are present in various inflamed tissues, driving immune cell activation and are associated with chronic inflammatory diseases, autoimmune diseases, and cancer. In the tumor environment, TLS promote immune cell infiltration into solid tumors, significantly correlating with survival in untreated patients (27–29).

In many cancers, a high density of TLS has been linked to prolonged patient survival (30–32). However, their clinical value remains limited. This study aimed to establish a more clinically valuable BC prognostic model based on TLS to provide more prognostic information for BC patients and guide treatment. We constructed and validated TLS in nine independent multicenter cohorts based on TLS genes associated with BC, combined with 108 machine learning algorithms. To determine the stability and predictive ability of AITS, we compared AITS with classical models and published models. The results of the nomogram confirmed that AITS, together with staging and age, accurately predicted overall survival (OS) in patients with different stages of BC. Additionally, patients with high AITS had a poorer prognosis and a higher frequency of recurrence compared with patients with low AITS.

Cancer is a group of diseases characterized by abnormal and uncontrolled cell growth caused by genetic mutations. These ‘drivers’ confer advantages to mutated cells over neighboring cells, affecting critical cellular functions. One major goal of cancer research is to discover these cancer-driver genes, identify targeted anticancer therapies, and find genomic biomarkers for prognosis and treatment (33). We examined the genomic alterations in AITS and found that patients with high AITS had higher TMB, diverse mutation characteristics, higher frequency of gene mutations, and more amplification and deletion of chromosome regions, suggesting a poor prognosis for this group. Notably, PVT1, MYC, CCD26, and GSDMC were more amplified at 8q24.21, while VANGL, TRIM45, TTF2, and VTCN1 were more deleted at 12P13.1 in high AITS BC patients. PVT1 is known to be abnormally expressed in several malignant tumors, including nasopharyngeal carcinoma, esophageal cancer, and colorectal cancer (34–36). PVT1 plays a critical role in BC proliferation, invasion, metastasis, and drug resistance in triple-negative BC (37–39). The MYC proto-oncogene produces transcription factors frequently activated in human tumors (40), and high GSDMC expression is associated with poor survival (41). Overexpression of TRIM45 can inhibit glioblastoma cell proliferation (42). These findings indirectly confirm that BC patients with high TLS have a poorer prognosis and reveal the underlying mechanisms.

Single-cell sequencing revealed that epithelial cells, T cells, B cells, macrophages, and mast cells predominated in BC tumor tissues. AITS mainly comprised T cells and B cells. Epithelial-mesenchymal transition (EMT) is crucial for embryonic development, tissue repair, and is present in many malignant tumors, including BC. Abnormal EMT marker expression is closely related to tumor invasion and metastasis (43, 44). We found that transcription factors involved in EMT play a role in TLS formation, with higher AITS activity in tumor aneuploid epithelial cells compared to tumor diploid and normal samples. This suggests that EMT may be a potential mechanism for AITS to predict BC. CellChat analysis revealed stronger cell-cell interactions and unique ligand-to-ligand communications in the low AITS group.

TME includes tumor cells, immune cells, extracellular matrix, fibroblasts, inflammatory cells, microvasculature, and signaling molecules (45). Remodeling the TME is crucial for improving clinical efficacy, making it a new target for modern tumor therapy (46). We assessed immune cell infiltration and immune checkpoint inhibitor (ICI) levels between different AITS subgroups. Less immune cell infiltration and fewer ICIs were activated in the TME of high AITS patients, suggesting that high AITS patients are more likely to be immunosuppressed and less responsive to ICI therapy, while low AITS patients are more likely to benefit from ICIs. Finally, we screened therapeutic targets and drugs to reveal chemotherapy effects among different patients. Our analyses identified five targets and several therapeutic agents, such as quizartinib, showing that high AITS patients were more susceptible to chemotherapy.

To address the ethical and legal concerns associated with AI-driven tools, particularly the reliance on automated CPT coding and the potential for misclassification, we have implemented several safeguards to ensure data accuracy and regulatory compliance. First, AI-generated CPT codes are subject to thorough human oversight and verification by experienced medical coders to minimize errors and enhance reliability. Additionally, a continuous model auditing and monitoring framework has been established to evaluate performance over time, detect biases, and recalibrate the model as needed. Our system strictly adheres to regulatory requirements, including HIPAA and GDPR, ensuring robust data privacy and security through encryption and secure data handling practices. To further enhance transparency and clinician trust, explainable AI (XAI) techniques have been integrated, providing interpretability of the model’s decision-making process. Furthermore, structured error handling protocols are in place to promptly address discrepancies, with escalation pathways for resolving conflicts between AI-generated and human-reviewed codes. Finally, comprehensive training programs are provided to healthcare professionals, equipping them with the necessary knowledge to effectively utilize AI-assisted coding tools while remaining aware of their limitations. These safeguards collectively contribute to maintaining the balance between automation efficiency and the critical need for human oversight and compliance with ethical and legal healthcare standards.

## Conclusion

Our TLS-based model outperforms traditional models, providing valuable insights into the tumor microenvironment and its role in cancer progression. This model enhances our understanding of BC biology and supports personalized therapeutic strategies, representing a significant advancement in personalized medicine. In conclusion, the TLS-based prognostic model is a powerful tool for predicting BC outcomes and tailoring treatment strategies, ultimately improving patient care and survival rates.

## Data Availability

All data used in this study were sourced from the public databases online or may be made available from the corresponding author upon reasonable request.
