# Supplementary material for: Development of a tertiary lymphoid structure-based prognostic model for breast cancer: integrating single-cell sequencing and machine learning to enhance patient outcomes
Source: Front Immunol. 2025 Feb 26;16:1534928. doi: 10.3389/fimmu.2025.1534928 (PMC11897234; doi:10.3389/fimmu.2025.1534928)
Supplement: Supplementary file 1 [file DataSheet1.pdf]

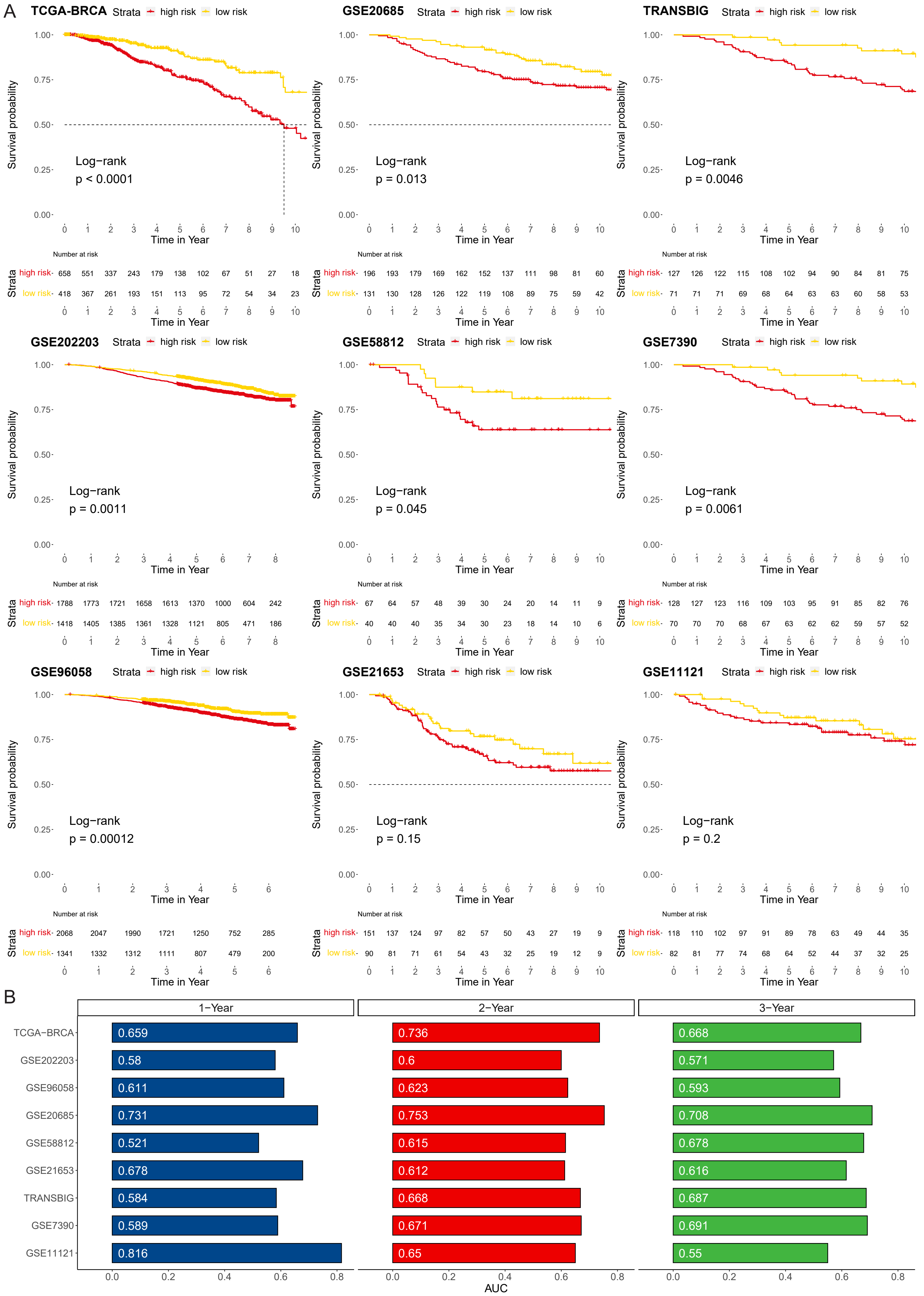

Figure S1. Kaplan-Meier survival analysis and time-dependent AUCs of the AITS model. (A) Kaplan-Meier survival curves for high- and low-risk groups in the training cohort (TCGA-BRCA) and validation cohorts (GSE20685, TRANSBIG, GSE202203, GSE58812, GSE7390, GSE96058, GSE21653, GSE11121). The high-risk group consistently showed higher mortality rates. (B) Time-dependent AUCs for the AITS model in the training cohort (TCGA-BRCA) and validation cohorts, demonstrating robust predictive performance at 1, 3, and 5 years.
