# Supplementary material for: Development of a tertiary lymphoid structure-based prognostic model for breast cancer: integrating single-cell sequencing and machine learning to enhance patient outcomes
Source: Front Immunol. 2025 Feb 26;16:1534928. doi: 10.3389/fimmu.2025.1534928 (PMC11897234; doi:10.3389/fimmu.2025.1534928)
Supplement: Supplementary file 2 [file DataSheet2.pdf]

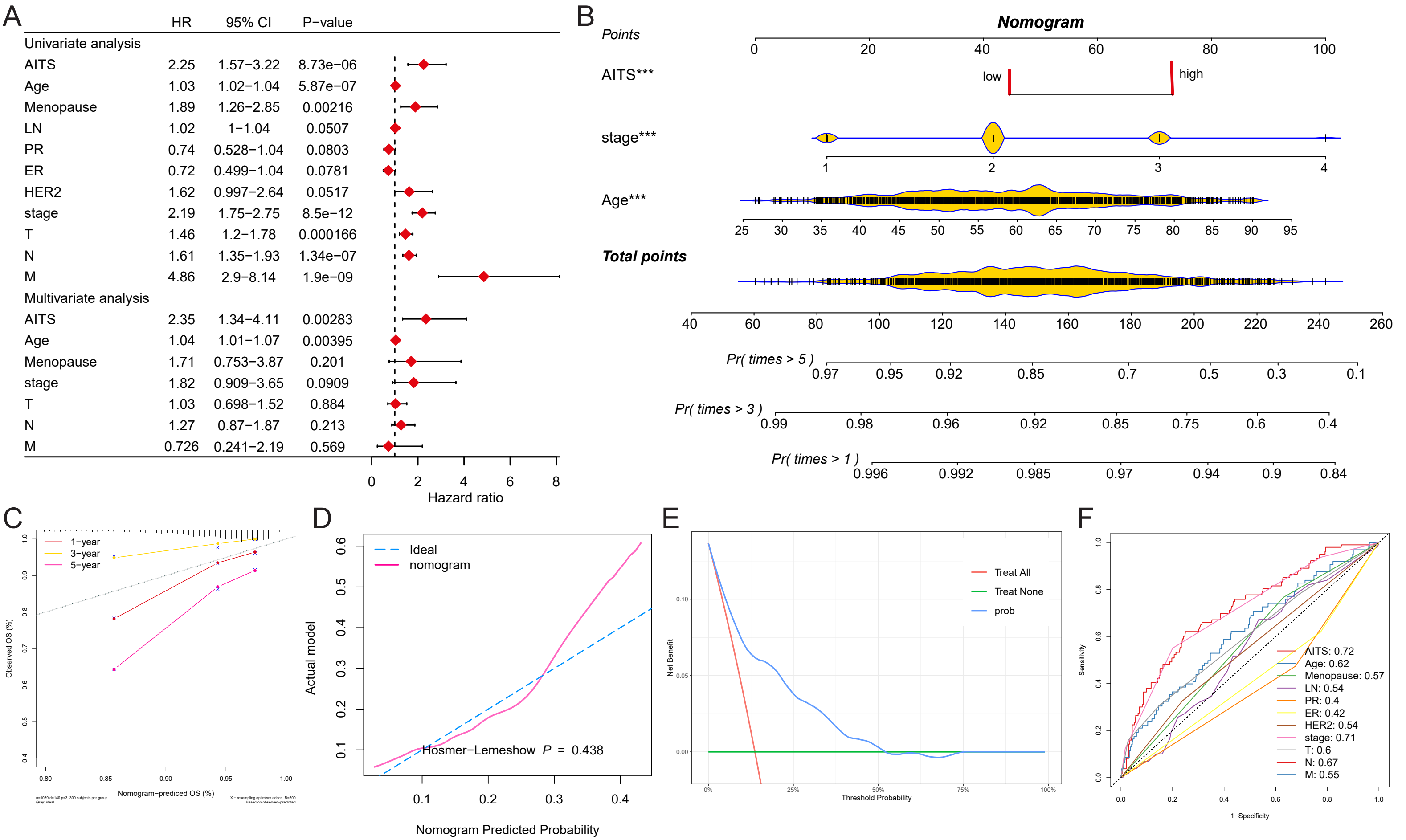

Figure S2. Cox regression analysis and nomogram for predicting survival. (A) Univariate and multivariate Cox regression analyses showing the independent prognostic value of the AITS model when adjusted for clinical and molecular variables. (B) Nomogram integrating AITS, pathological stage, and age for predicting one-, three-, and five-year survival probabilities in breast cancer patients. (C) Calibration curve indicating the high accuracy of the nomogram. (D) Hosmer-Lemeshow test showing no significant difference between predicted and observed survival probabilities. (E) Decision curve analysis demonstrating the net benefit of the AITS model compared to treating all or no patients. (F) ROC curves comparing the prognostic accuracy of the AITS model with other clinical pathological factors.
