# Supplementary material for: Development of a tertiary lymphoid structure-based prognostic model for breast cancer: integrating single-cell sequencing and machine learning to enhance patient outcomes
Source: Front Immunol. 2025 Feb 26;16:1534928. doi: 10.3389/fimmu.2025.1534928 (PMC11897234; doi:10.3389/fimmu.2025.1534928)
Supplement: Supplementary file 4 [file DataSheet4.pdf]

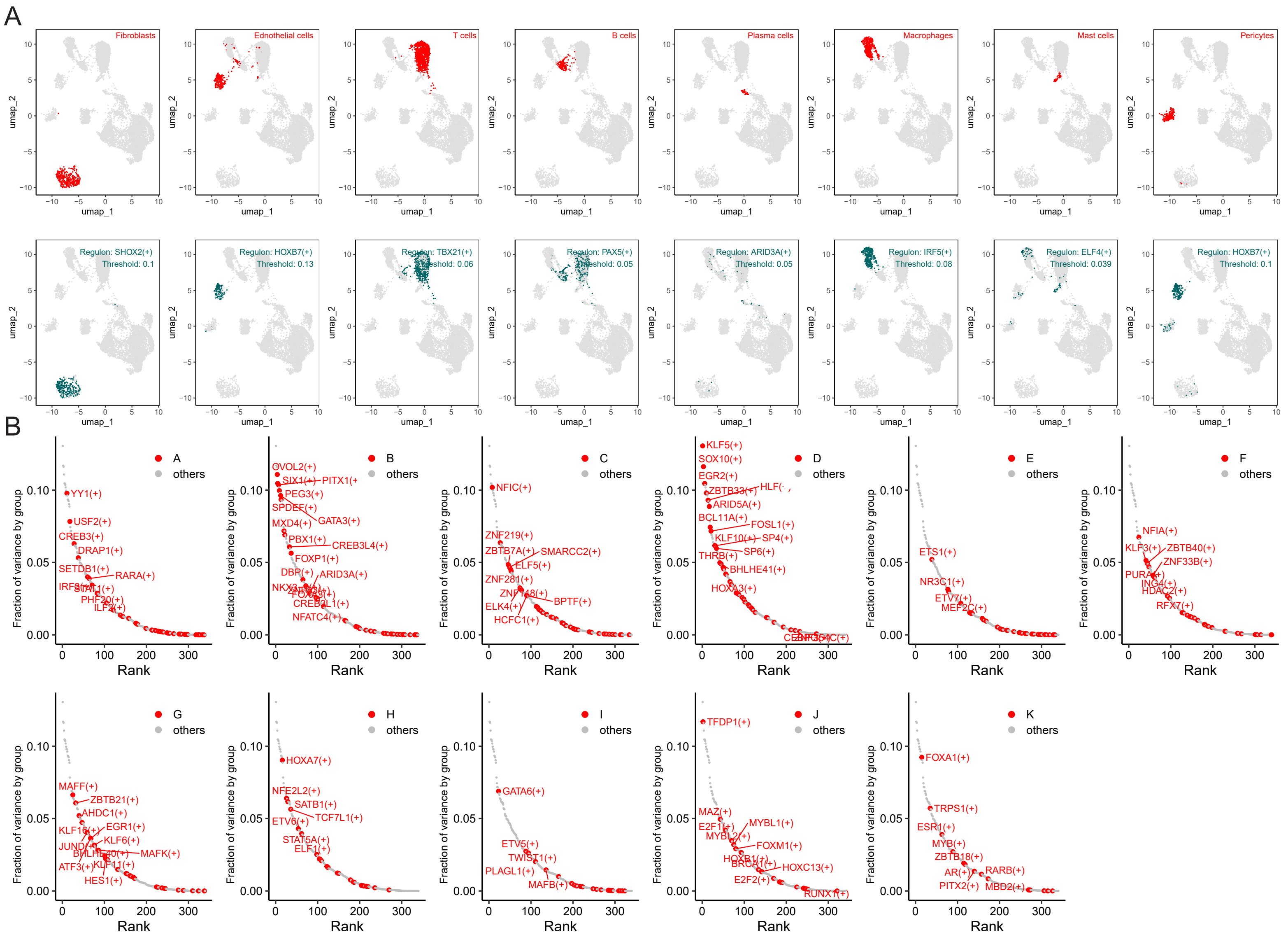

Figure S4. Detailed transcriptional regulation analysis. (A) UMAP projections categorize samples by transcription factor activity, revealing distinct cell types based on their transcriptional profiles, aiding in the identification of unique cellular behaviors and properties. (B) Illustrates the contribution of different transcription factor groups to AITS, with significant transcription factors highlighted and ranked based on their RSS, showcasing their regulatory impact on gene expression particularly in epithelial cells.
