# Supplementary material for: Development of a tertiary lymphoid structure-based prognostic model for breast cancer: integrating single-cell sequencing and machine learning to enhance patient outcomes
Source: Front Immunol. 2025 Feb 26;16:1534928. doi: 10.3389/fimmu.2025.1534928 (PMC11897234; doi:10.3389/fimmu.2025.1534928)
Supplement: Supplementary file 3 [file DataSheet3.pdf]

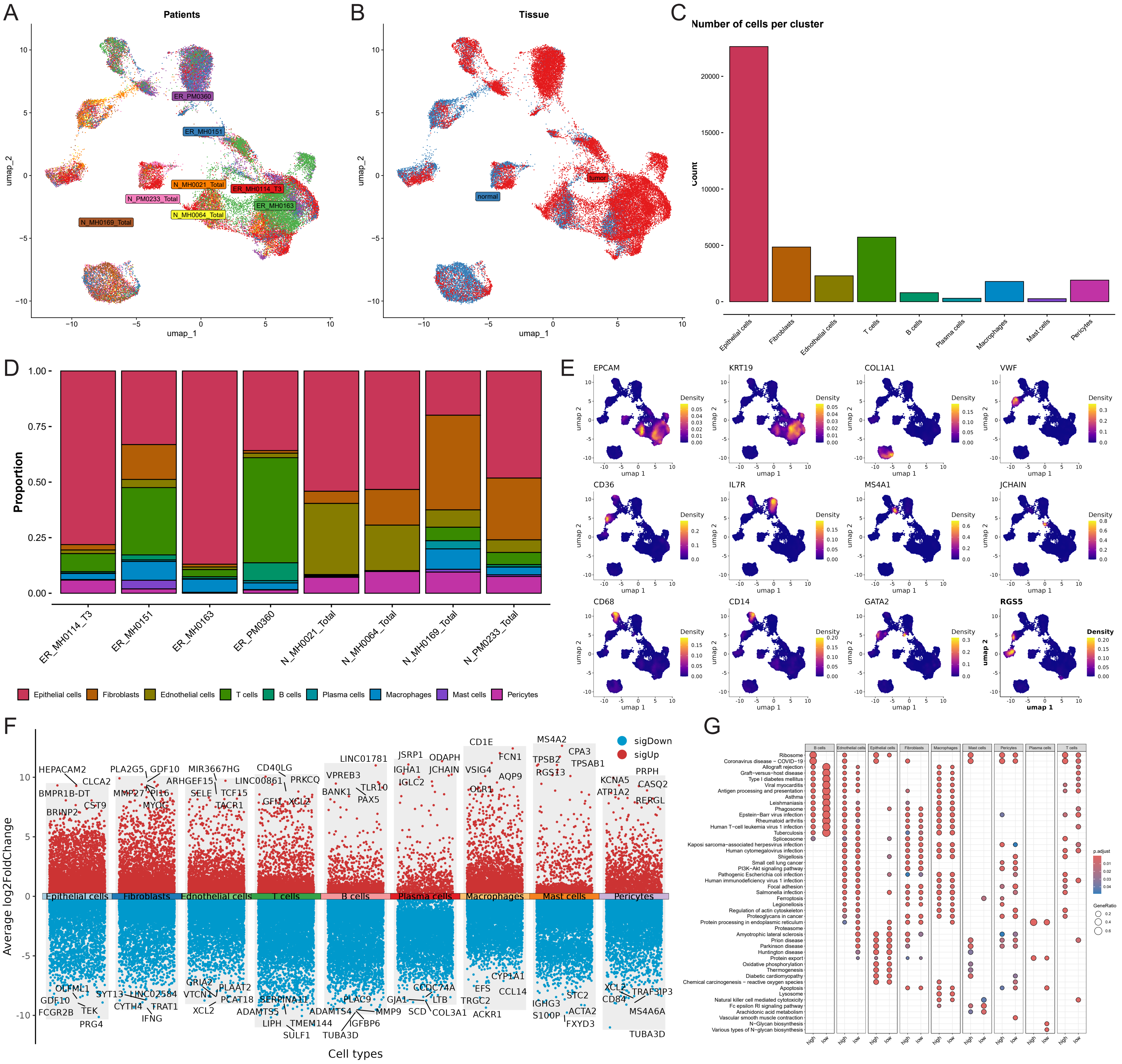

Figure S3. Single-cell transcriptome analysis details. (A) Clustering of cells from eight BC patients. (B) Classification of cells from tumor and normal tissues. (C) Summary of cell type numbers and proportions across patients. (D) Proportion of each cell type in tumor versus normal tissues. (E) Density plots for representative markers of each cell type. (F) Differential expression analysis results. (G) GSEA analysis revealing functional pathways associated with high and low AITS.
